# Supplementary material for: Enhanced prediction of thrombotic events in hospitalized COVID-19 patients with soluble thrombomodulin
Source: PLoS One. 2025 Mar 19;20(3):e0319666. doi: 10.1371/journal.pone.0319666 (PMC11922281; doi:10.1371/journal.pone.0319666)
Supplement: S1 File — (DOCX) [file pone.0319666.s001.docx]

**Supplementary Figure S1. Local protocol for managing COVID-19 hospital admitted patients with worsening condition.**

**
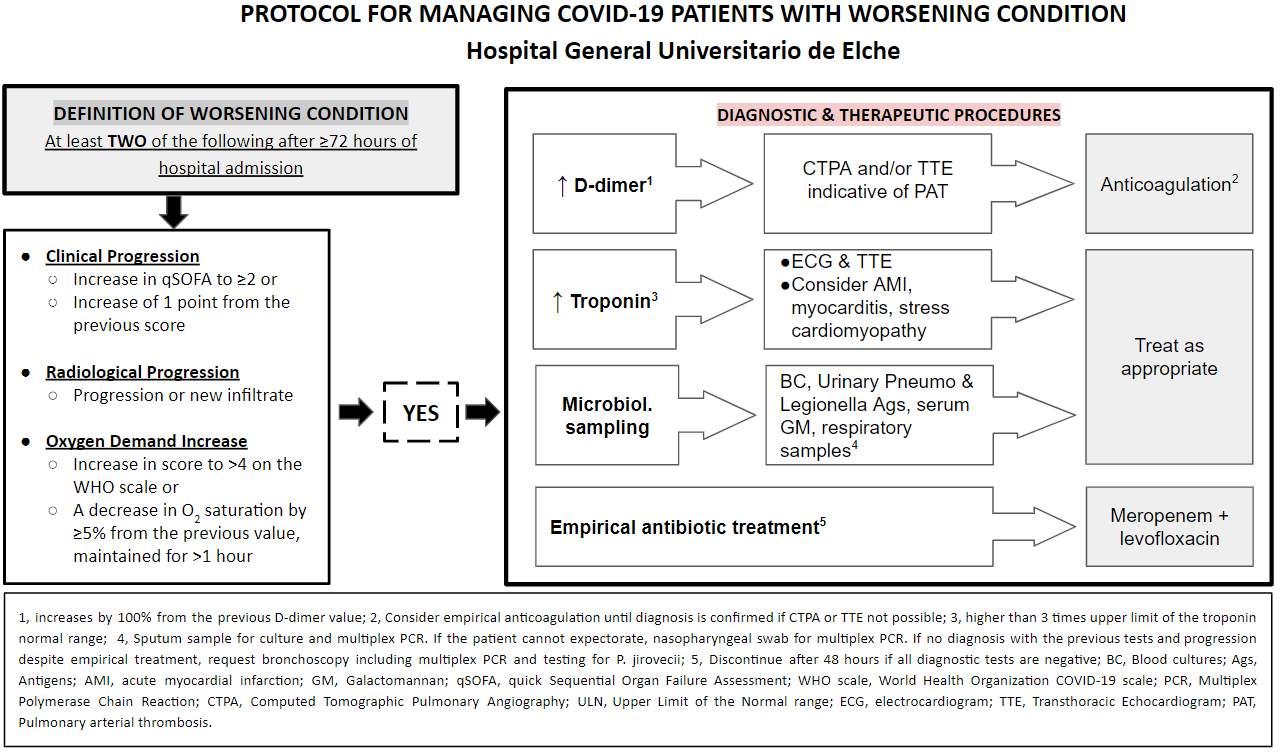
**

**Supplementary Table S1. Baseline predictors of 28-day thrombotic events in the entire cohort.**

|  |  | **Thrombotic events** | |  |
| --- | --- | --- | --- | --- |
|  | **All** | **No** | **Yes** | ***P*-value** |
| **N** | 2524 | 2451 | 73 | - |
| **Female sex** | 1092 (43) | 1060 (43) | 32 (44) | 0.999 |
| **Age, years** | 67 (54, 80) | 67 (54, 80) | 72 (61, 79) | 0.121 |
| **Charlson Comorbidity Index** | 3 (1, 5) | 3 (1, 5) | 4 (2, 5) | 0.021 |
| **Any comorbidity** | 1892 (75) | 1631 (67) | 61 (84) | 0.139 |
| **Cardiovascular disease** | 849 (34) | 821 (33) | 28 (38) | 0.490 |
| **Hypertension** | 1169 (46) | 1133 (46) | 36 (49) | 0.735 |
| **Diabetes** | 622 (25) | 606 (25) | 16 (22) | 0.653 |
| **WHO COVID-19 severity score (5-6-7)** | 96 (4) | 84 (3) | 12 (16) | 0.001 |
| **FiO2 (%), log10** | 28 (24, 32) | 28 (24, 32) | 28 (28, 36) | 0.083 |
| **eGFR (mL/min/1.73 m²), log10** | 91.8 (65.8, 105) | 91.8 (65.8, 105) | 89.5 (64.5, 98) | 0.045 |
| **C-reactive protein (mg/dL), log10** | 44 (16.6, 93.4) | 44 (16.7, 93) | 44 (9.9, 113) | 0.821 |
| **IL-6 (pg/mL), log10** | 33 (9.6, 118.7) | 33 (9.4, 117) | 35.5 (18.5, 222) | 0.058 |
| **Ferritin (**ng/dL)**, log10** | 269 (124, 556) | 269 (123, 555) | 291 (134, 586) | 0.606 |
| **LDH (**U/L)**, log10** | 246 (201, 315) | 246 (201, 313) | 275 (229, 426) | 0.001 |
| **NLR** | 4.5 (2.8, 7.9) | 4.4 (2.8, 7.8) | 6.1 (4, 12.4) | 0.001 |
| **D-Dimer, μg/mL** | 0.7 (0.4, 1.4) | 0.7 (0.4, 1.4) | 2.3 (0.8, 7.4) | 0.001 |
| **INR** | 1.1 (1.0, 1.2) | 1.1 (1.0, 1.2) | 1.1 (1, 1.2) | 0.968 |
| **Platelets, (**x 10^3^/μL)**, log10** | 186 (146, 238) | 186 (146, 238) | 214 (169, 289) | 0.002 |
| **RT-PCR Cycle threshold** | 25 (19, 31) | 25 (19, 31) | 29 (22, 33) | 0.012 |
| **Clinical events at 28 days** |  |  |  |  |
| Overall mortality | 237 (9) | 226 (9) | 11 (15) | 0.138 |
| Mechanical ventilation | 148 (6) | 133 (5) | 15 (21) | 0.001 |
| NIV or HFO | 211 (8) | 192 (8) | 19 (26) | 0.001 |
| ICU admission | 207 (8) | 181 (7) | 26 (36) | 0.001 |

Data are presented as percentages for categorical variables and medians with interquartile ranges for continuous variables. WHO, World Health Organization; FiO2, fraction of inspired oxygen; eGFR, estimated glomerular filtration rate; IL-6, interleukin 6; LDH, lactate dehydrogenase; NLR, neutrophil-to-lymphocyte ratio; INR, International Normalized Ratio; RT-PCR, reverse transcriptase polymerase chain reaction; NIV, non-invasive ventilation; HFO, high-flow oxygen; ICU, intensive care unit.

**Supplementary Table S2. Incidence rates for venous and arterial thrombotic events at various time points and patient subgroups.**

|  | **Cases** | **Follow-up (PD)** | **IR (95%CI) x 1000 PD** |
| --- | --- | --- | --- |
| **Thrombotic events** |  |  |  |
| 28 days | 73 | 63560 | 1.15 (0.90 - 1.44) |
| 60 days | 86 | 124057 | 0.69 (0.55 - 0.86) |
| 90 days | 93 | 174981 | 0.53 (0.42 - 0.65) |
| During hospital stay | 61 | 18742 | 3.25 (2.49 - 4.18) |
| **Sex** |  |  |  |
| male | 41 | 36198 | 1.13 (0.81 - 1.16) |
| female | 32 | 27225 | 1.18 (0.80 - 1.66) |
| **Any comorbidity** |  |  |  |
| No | 12 | 16168 | 0.74 (0.38 - 1.29) |
| yes | 61 | 47074 | 1.29 (0.99 - 1.66) |
| Cardiovascular disease | 28 | 20915 | 1.34 (0.89 - 1.93) |
| Hypertension | 36 | 28866 | 1.25 (0.87 - 1.73) |
| Diabetes | 16 | 15451 | 1.05 (0.59 - 1.68) |
| **WHO COVID-19 severity score** |  |  |  |
| 3 | 29 | 29990 | 0.97 (0.64 - 1.39) |
| 4 | 32 | 31342 | 1.02 (0.69 - 1.44) |
| 5 | 8 | 1036 | 7.7 (3.33 - 15.22) |
| 6 | 2 | 594 | 3.37 (0.41 - 12.16) |
| 7 | 2 | 461 | 4.34 (0.53 - 15.67) |
| **SARS-CoV-2 variant** |  |  |  |
| all (n=2524) |  |  | - |
| Ancestral | 29 | 31767 | 0.91 (0.61 - 1.31) |
| Alfa | 28 | 12912 | 2.17 (1.44 - 3.13) |
| Delta | 10 | 5460 | 1.83 (0.88 - 3.37) |
| Omicron | 6 | 12934 | 0.46 (0.17 - 1.01) |
| **Clinical events at 28 days** |  |  |  |
| overall mortality | 11 | 2736 | 4.02 (2.01 - 7.19) |
| mechanical ventilation | 15 | 3508 | 4.27 (2.39 - 7.05) |
| NIV or HFO | 9 | 4712 | 4.03 (2.43 - 6.29) |
| ICU admission | 26 | 4683 | 5.22 (3.41 - 7.65) |

PD, person-days; IR, incidence rate; CI, confidence interval; NIV, non-invasive ventilation; HFO, high-flow oxygen; ICU,intensive care unit.

**Supplementary Figure S2. Stacked bar graph illustrating the frequency (%) of venous and arterial thrombotic events across different time periods post-hospital admission.**


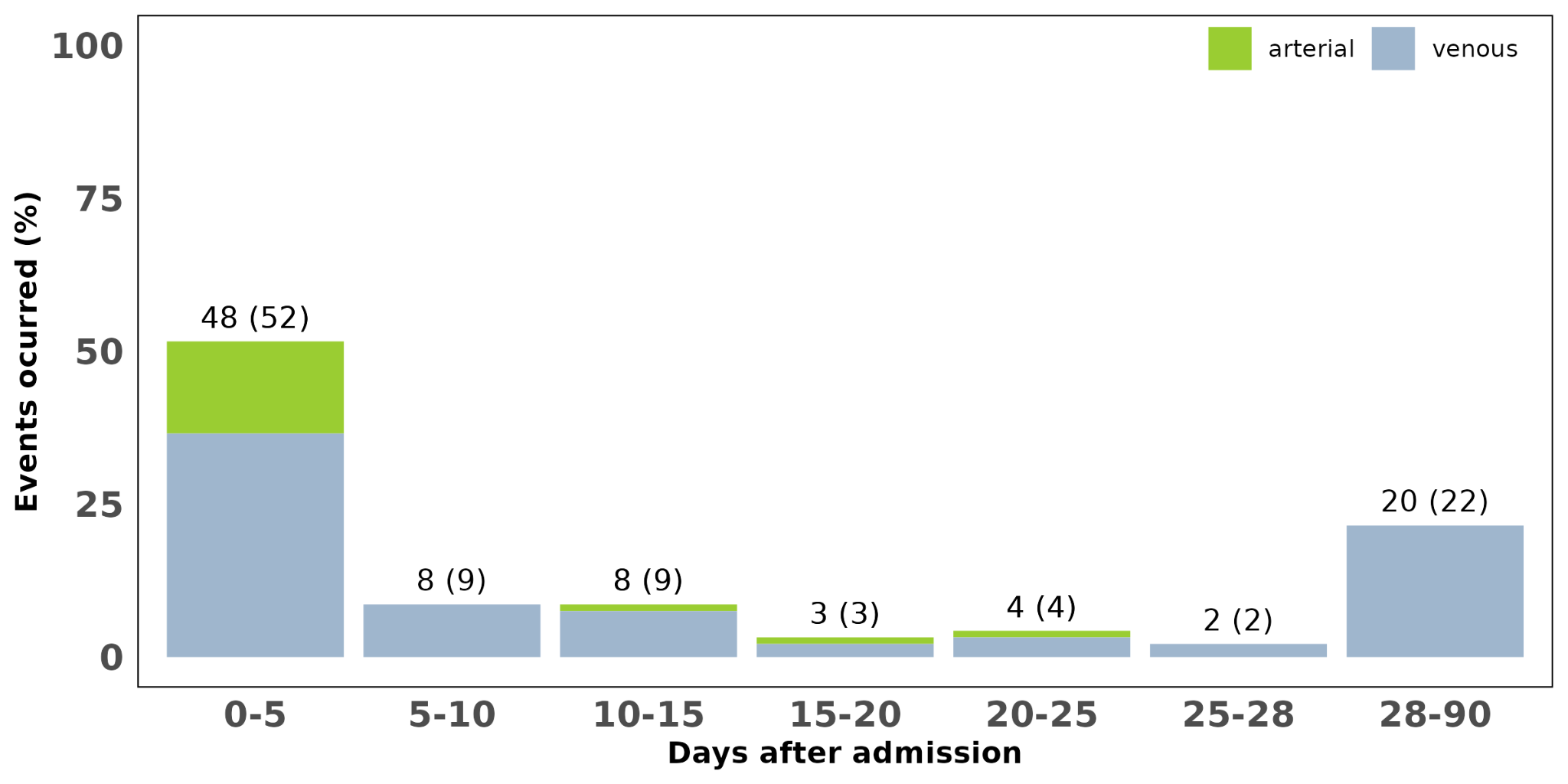


**Supplementary Table S3. Characteristics of thrombotic events.**

|  | **Value** | |
| --- | --- | --- |
| **Number of events** | 73 |  |
| **Days of symptoms**, median (Q1, Q3) | 5 | (3-8) |
| **TE type** | - |  |
| 1) ***Venous thrombosis*** | 56 | (77%) |
| 1.1) PAT | 46 |  |
| PESI category (Risk 30-day mortality) | | |
| I (0-1.6%) | 3 |  |
| II (1.7-3.5%) | 10 |  |
| III (3.2-7.1%) | 12 |  |
| IV (4.0-11.4%) | 13 |  |
| V (10-24.5%) | 8 |  |
| CTPA features |  |  |
| Central | 11 |  |
| Peripheral | 35 |  |
| 1.2) DVT without PAT | 10 |  |
| 2) ***Arterial thrombosis**** | 17 | (23%) |

TE, thrombotic events; PAT, pulmonary arterial thrombosis; PESI, Pulmonary Embolism Severity Index (Jacques Donzé, Grégoire Le Gal, Michael J Fine et al. Prospective validation of the Pulmonary Embolism Severity Index. A clinical prognostic model for pulmonary embolism. Thromb Haemost. 2008 Nov;100(5):943-8); Q1, first Quartile; Q3, third Quartile; CTPA, computed tomographic pulmonary angiography; DVT, deep venous thrombosis; *, Nine ischaemic strokes, six acute myocardial infarctions and two acute peripheral ischaemia cases.

**Supplementary Figure S3. SMD changes in matching variables following propensity score.**


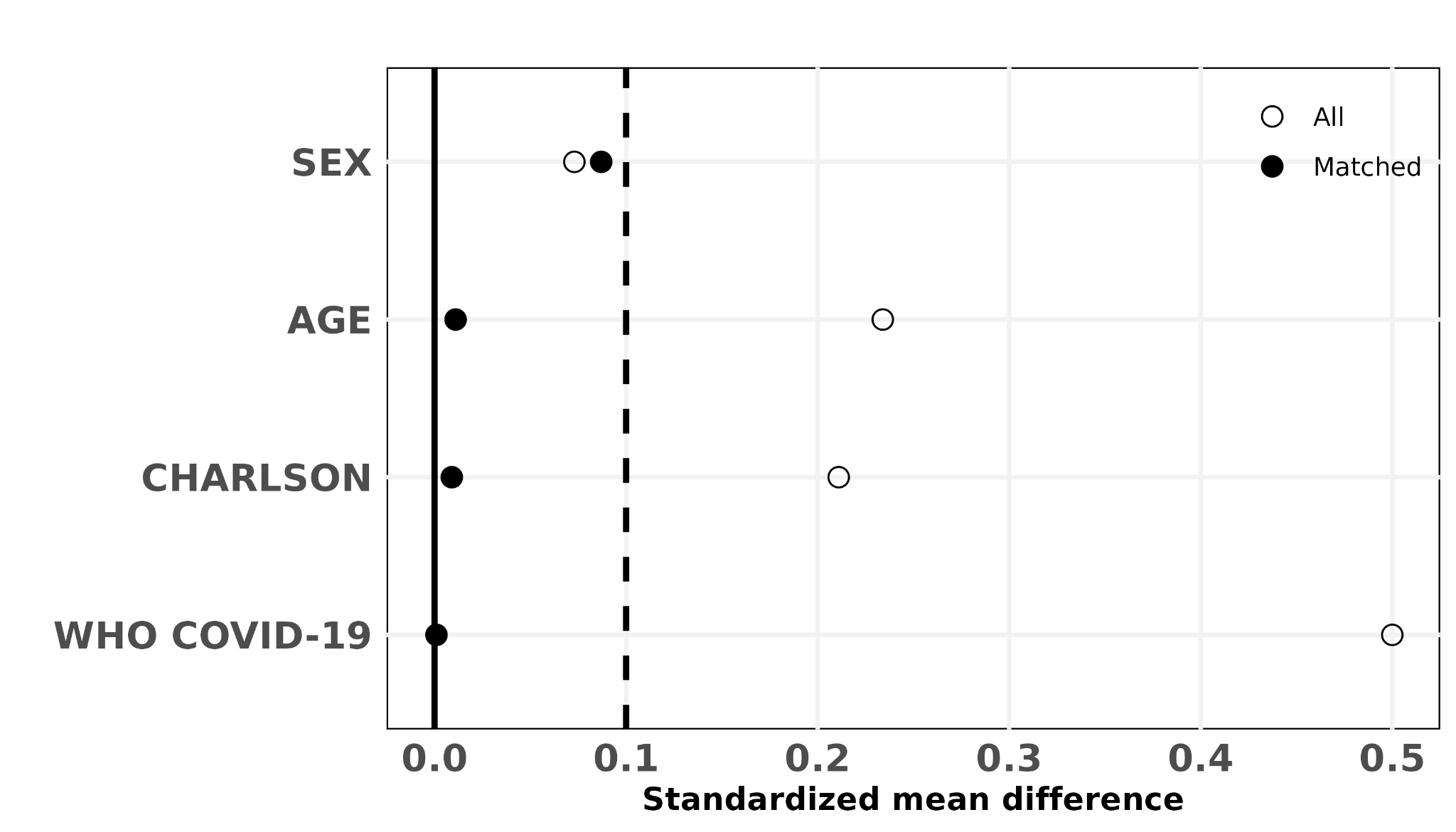


SMD, standardized mean difference; Charlson, Charlson Comorbidity Index; WHO COVID-19, World Health Organization COVID-19 Ordinal Scale. This figure illustrates SMD for the variables used in the propensity score matching process: sex, age, Charlson comorbidity index, and WHO COVID-19 severity score. Open circles represent the SMD before matching ("All"), while filled circles represent the SMD after matching ("Matched"). The dashed line at 0.1 indicates the commonly accepted threshold for adequate balance. Values closer to 0 indicate better balance between cases and controls. The results demonstrate that the matching process significantly reduced imbalances in all variables, achieving acceptable balance across the matched cohort.

**
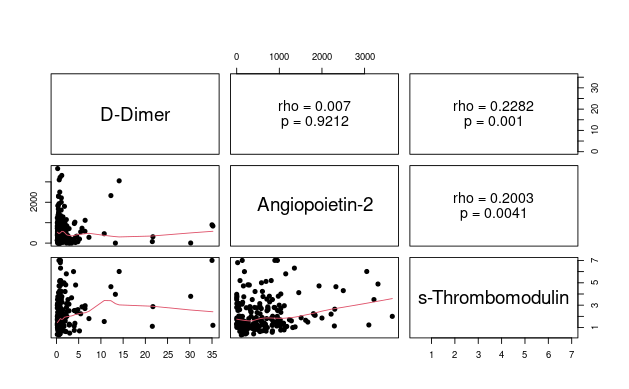
Supplementary Figure S4. Correlations between the three coagulation biomarkers studied.**

Value in cells represents Pearson's R. **, *p*-value between 0.05 and 0.001. Scatter plot represents the individual data points, and the red line is the regression fit.

**Supplementary Table S4. Correlation between the coagulation parameters and other plasma biomarkers.**

|  | **D-dimer**  **(μg/mL)** | **Thrombomodulin**  **(ng/mL)** | **Angiopoietin-2**  **(pg/mL)** |
| --- | --- | --- | --- |
| **Age** | 0.02 | 0.31*** | 0.20** |
| **Charlson Comorbidity Index** | 0.07 | 0.43*** | 0.24*** |
| **IL-6,** pg/mL | -0.02 | 0.17* | 0.02 |
| **C-reactive protein,** mg/dL | 0.04 | 0.19** | 0.14* |
| **Serum creatinina** | 0.01 | 0.44*** | 0.23*** |
| **Ferritin,** ng/dL | 0.06 | 0.22*** | -0.01 |
| **Lactate Dehydrogenase,** U/L | 0.21** | 0.24*** | -0.04 |

IL-6, interleukin 6. *, *p*-value < 0.05; **, *p*-value between 0.05 and 0.001; ***, *p*-value < 0.001.

**Supplementary Figure S5. Multivariate Cox proportional hazards regression analysis of baseline predictors for 28-Day venous thrombotic events in the propensity score cohort.,**

**
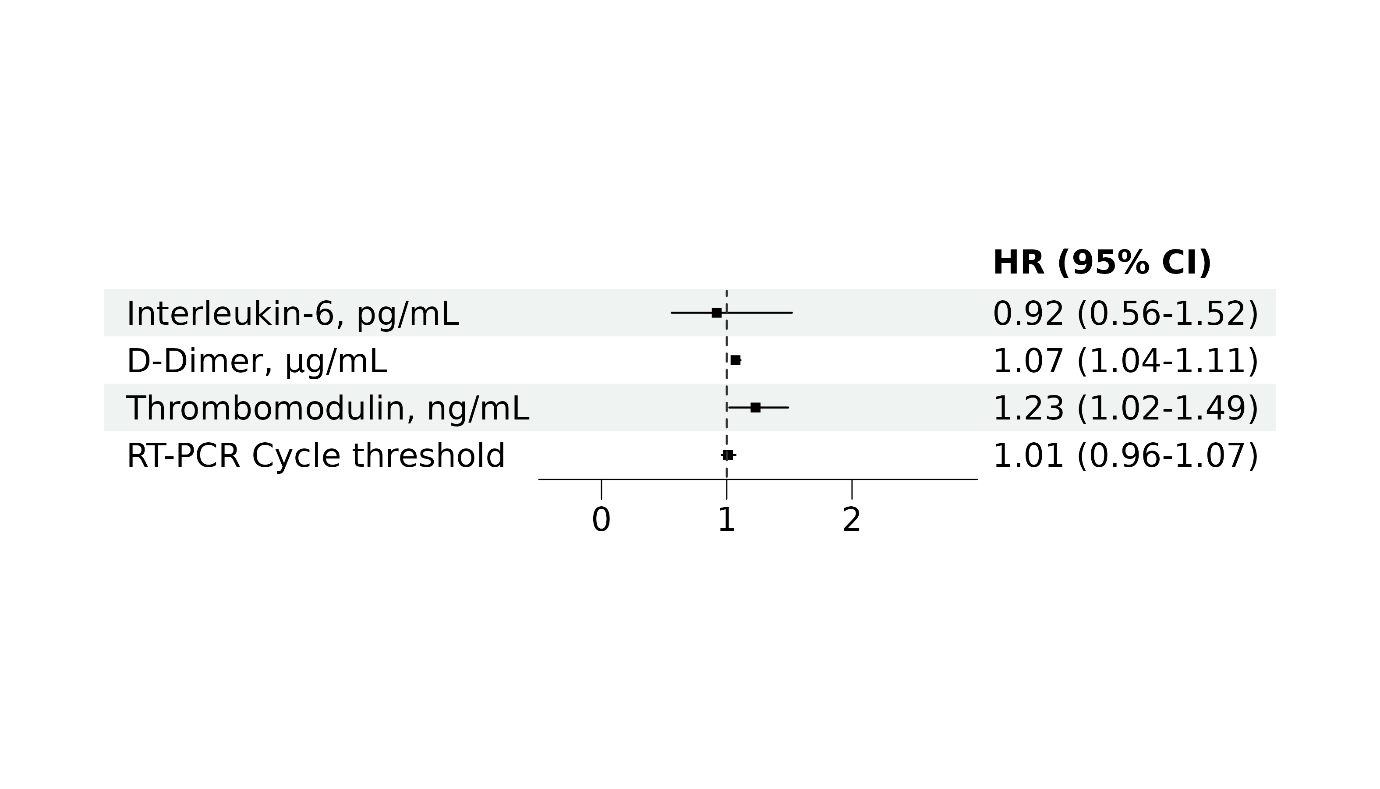
**

HR, Hazard ratio; CI, confidence interval; RT-PCR, reverse transcriptase polymerase chain reaction for SARS-CoV-2.
