# Supplementary material for: Enhanced prediction of thrombotic events in hospitalized COVID-19 patients with soluble thrombomodulin
Source: PLoS One. 2025 Mar 19;20(3):e0319666. doi: 10.1371/journal.pone.0319666 (PMC11922281; doi:10.1371/journal.pone.0319666)
Supplement: Attachment — Submitted filename: Reviewers response.docx [file pone.0319666.s003.docx]

Reviewers' comments: 
Reviewer's Responses to Questions

**REVIEWER #1:**

**This is a nested case control study of patients with COVID-19 and examined the utility of sTM and ang2 as predictive markers for TE.**

**It is well executed and well written study, however, there are some major shortcomings that directly impact the scope of this work.**

**In the introduction, authors have mentioned the limitations of D dimer and that it is non specific for thrombosis. I would argue the same is true for soluble thrombomodulin. It is a marker of endothelial injury, and as a result can be elevated at the time of thrombosis, multi-organ injury, respiratory failure, etc.**

Response: We agree that soluble thrombomodulin (sTM) is a marker of endothelial injury and acknowledge that its elevation is not specific to thrombosis but can occur in a variety of clinical conditions, including multi-organ injury and respiratory failure. To address this important point, we have revised the introduction to explicitly discuss the non-specific nature of sTM and the broader context of endothelial dysfunction. These revisions aim to provide a more balanced discussion of the limitations and interpretative considerations associated with sTM as a predictive marker for thrombotic events in COVID-19.

Changes made in the manuscript: Specifically, we have modified the fourth paragraph of the introduction (line 80) to highlight that sTM can be elevated in various other processes involving endothelial injury. The revised sentence now reads (The changes are highlighted for clarity):

“Although soluble thrombomodulin (sTM) can be elevated in various other processes involving endothelial injury, research suggests that one…”

**Authors mentioned about routine CTPA to rule out PAT. How about 4 extremity doppler ultrasound? If that was not done routinely with CTPA, is there a chance that an extremity DVT was missed and people were inappropriately assigned to the no thrombosis category? Please clarify.**

Response: We appreciate the reviewer’s insightful comment regarding the possibility of undetected deep venous thrombosis (DVT) in patients who underwent only routine computed tomography pulmonary angiography to rule out pulmonary artery thrombosis. In our study, four-extremity Doppler ultrasound was not conducted on a routine basis. Instead, doppler ultrasonography of the lower (and, when clinically warranted, upper) extremities was performed at the discretion of the treating physician, primarily in cases of suspected DVT. We acknowledge that this targeted approach may have left some asymptomatic or otherwise unrecognized DVT cases undiagnosed.

Changes made in the manuscript: Specifically, we have modified the Methods and Discussion sections to clarify our imaging approach for diagnosing DVT. The revised sentences now read:

Methods (paragraph 2, line 104)

“…thrombosis (PAT). Four-extremity doppler ultrasound was not routinely performed but was used at the physician’s discretion for suspected deep venous thrombosis (DVT). If CTPA was not feasible, the…”

Discussion (paragraph 4, line 338)

“…prophylaxis and early detection of high risk patients, which included serial D-dimer measurements [25]. Additionally, our use of targeted, rather than universal, four-extremity duplex ultrasound may have missed asymptomatic DVT, leading to a lower observed incidence. Nevertheless, D-dimer, primarily…”

**Authors acknowledge that D dimer is a not a great marker for arterial thrombosis in their discussion. It will be helpful to compare ROC curves for D dimer with and without sTM to truly understand if indeed sTM is a better predictor for arterial thrombosis than D dimer or not.**

Response: We appreciate the reviewer’s suggestion. Indeed, we performed such an analysis (see Figure below); however, the number of arterial events in our dataset was quite small, which limited our statistical power. Consequently, the ROC curves for sTM and D-dimer in this subgroup appeared very similar, making it difficult to draw firm conclusions about sTM’s comparative predictive ability for arterial thrombosis. We will add a clarifying statement to the Discussion in the revised manuscript, acknowledging that while sTM may help refine risk assessment in venous thrombotic events, the current sample size for arterial events does not permit definitive conclusions regarding sTM’s superiority over D-dimer.

Changes made in the manuscript:

Discussion (paragraph 6, line 369)

“… misclassification. Furthermore, biomarker measurements were taken at different times for thrombotic cases and controls. While this reflects real-world clinical practice, it may introduce temporal bias, as elevated levels in cases could partly reflect the thrombotic process rather than purely pre-event risk. Finally, the low number of arterial events precluded subgroup analyses, limiting the conclusions that can be drawn from these specific outcomes. The strengths…”

**REVIEWER #2: The authors measured serum levels of sTM, Angiopoietin-2 and D-Dimers in 73 patients developing thrombotic events (TE) within 28 days of hospital admission and 297 patients without TE.**

**1.- The Biomarkers were measured closest to TE in the 73 positives, but at hospital admission for the other patients. This does not work. The analysis can either aim to be predictive, then all measurements have to take place at hospital admission. Or the analysis is diagnostic, then all measurements need to take place on matched days after hospital admission, or else a time course can be shown for all patients from hospital admission to days 5 - 10 - 28 comparing patients with and without TE.**

Response: We appreciate the reviewer’s insightful observation regarding the importance of baseline values for demonstrating the predictive capacity of soluble thrombomodulin (sTM) and D-dimer. Our study design, based on measurements starting from the time of hospital admission rather than at symptom onset or infection, reflects a pragmatic, real-world scenario of patient management. However, this pragmatic approach, where samples are taken at different time points, inherently blends predictive and diagnostic information, making it challenging to isolate purely predictive biomarkers. However, as shown in the supplementary figure S2, the occurrence of approximately two-thirds of thrombotic events within the first five days of hospital admission may help mitigate, though not eliminate, this potential bias. We have addressed this issue in the limitations section and we will ensure that the terminology used throughout the manuscript is precise to accurately convey the scope and constraints of our findings.

Changes made in the manuscript: We have revised the Discussion section to clarify the timing of biomarker measurements and its implications for the study design. The updated sentences now state:

Discussion (first paragraph, lines 303-310):

“This study aimed to identify new plasma biomarkers for thrombotic events in hospitalized COVID-19 patients, with greater potential to refine risk assessment compared to ~~better predictive capacity than~~ D-dimer alone, to enable personalized prophylaxis for people at higher risk. Notably, sTM levels emerged as an independent marker that can improve the ability ~~enhancing the predictive accuracy~~ of D-dimer to identify patients at higher risk of ~~for~~ venous and arterial events. In particular, the inclusion of sTM reduced the false positive rate associated with D-dimer alone. Angiopoietin-2 concentration correlated with sTM concentration but showed no association with thrombotic events or mortality, nor dit it add to the risk stratification for ~~and did not improve the prediction of~~ such events.”

Discussion (sixth paragraph, line 365):

“…leading to potential misclassification. Furthermore, biomarker measurements were taken at different time-points for thrombotic cases and controls, reflecting a real-world approach that captures data near the event but deviates from strictly predictive or diagnostic designs. This may introduce temporal bias, as elevated levels in cases could partly reflect the thrombotic process rather than pre-event risk. The strengths…”

**2.- The Discussion emphasizes the predictive nature of the results, then values measured at hospital admission have to be shown, but removal of the 20 patients with TEs after day 28 is not justifiable.**

Response: As mentioned in our response to the reviewer’s previous comment, we have revised the manuscript to moderate the language used, ensuring it more accurately reflects the pragmatic nature of our study design. Specifically, we have clarified that the study is not strictly predictive but rather aims to explore associations between biomarkers and thrombotic events in a real-world clinical context, where biomarker measurements were not uniformly timed across all patients. These limitations are now explicitly acknowledged in the discussion section.

We understand the reviewer’s concern regarding the exclusion of thrombotic events beyond day 28. Our rationale was to focus on the acute phase of SARS-CoV-2 infection, where the influence of COVID-19–related pathophysiology on thrombosis is most pronounced, and where biomarkers like sTM are known to reflect acute endothelial injury more effectively. Including later events could introduce confounding factors, such as prolonged immobilization or comorbidities, which might dilute the association between biomarker levels and thrombotic risk in the early phase. While we acknowledge that this decision may limit the generalizability of our findings, we believe it strengthens the internal validity for investigating the acute effects of COVID-19 on thrombosis.

**3.- This correction of timing should also include all lab values (Table 1).**

Response: We appreciate the reviewer’s observation regarding the timing of lab values in Table 1. We confirm that all values listed in the table correspond to measurements taken at hospital admission, except for D-dimer and sTM, which were determined at the time points previously indicated in the manuscript. To address this, we have added a note to the foot of Table 1 to clarify this aspect. Unfortunately, we do not have the capability to perform these determinations at additional time points.

Changes made in the manuscript:

Table 1 footnote:

“*Soluble Thrombomodulin and Angiopoietin-2 plasma levels were determined in the subset of individuals with available samples (N=219 [176 without events and 43 with thrombotic events]). Data are presented as percentages for categorical variables and medians with interquartile ranges for continuous variables. WHO, World Health Organization; FiO2, fraction of inspired oxygen; eGFR, estimated glomerular filtration rate; IL-6, interleukin 6; LDH, lactate dehydrogenase; NLR, neutrophil-to-lymphocyte ratio; s-TM, soluble Thrombomodulin; INR, International Normalized Ratio; RT-PCR, reverse transcriptase polymerase chain reaction; NIV, non-invasive ventilation; HFO, high-flow oxygen; ICU, intensive care unit; OR, odds ratio. All values were measured at hospital admission except for D-dimer and sTM, which were determined closest to the thrombotic event in cases (prioritizing pre-event samples) and at the earliest available sample after admission in controls, as specified in the Methods section.”

**4.- Multivariate logistic regressions include D-Dimers, sTM, IL-6 and RT-PCR-cycle threshold. I cannot understand this selection - either include clinically meaningful variables (age, sex, comorbidity etc.) or else include all variables significant in univariable logistic regression from Table 1.**

Response: Thank you for pointing this out. We appreciate the opportunity to clarify our approach to variable selection for the multivariate logistic regression models. IL-6 was added as a key inflammatory mediator in COVID-19-associated coagulopathy, and RT-PCR cycle threshold was included as a proxy for viral load, which is linked to disease severity and endothelial dysfunction.

Clinical variables such as age, sex, Charlson comorbidity index, and WHO COVID-19 severity score were incorporated into the propensity score matching to ensure balanced cohorts of cases and controls. This approach accounted for these factors without reintroducing them into the regression models, avoiding collinearity and over-adjustment. Additionally, not all univariable predictors from Table 1 were included to prevent overfitting, given the relatively small number of events. Instead, we focused on variables with strong biological plausibility and mechanistic links to thrombotic risk.

Changes made in the manuscript:

Methods (Statistical analyses section, line 167):

“The main analyses involved logistic regression modeling. We included potential confounders in the models, including baseline variables selected based on statistical and clinical significance. Variables used for propensity score matching (age, sex, Charlson comorbidity index, and WHO COVID-19 severity score) were not reintroduced in the models to avoid collinearity and over-adjustment. To enhance….”

**5.- Figures 1 and 2: TE until day28? Overall mortality is a time dependent event, Cox regression should be used.**

Response: Thank you for your observation regarding Figures 1 and 2. We confirm that the thromboembolic events (TE) shown in the figures are limited to those occurring within the first 28 days after hospital admission.

We thank the reviewer for suggesting the use of Cox regression, which is more appropriate for the analysis of overall mortality in this context. In response, we have reanalyzed the data using a Cox proportional hazards model. The results remain consistent with our initial findings, and we have updated the Methods, Results, and Figures 2, 3 and S5 accordingly.

Changes made in the manuscript:

Figure 1 Title:

“Figure 1. Boxplots illustrating soluble thrombomodulin (Panel a) and D-dimer (Panel b) plasma concentration in relation to mortality and thromboembolic events within 28 days.”

Methods (“Statistical analyses” section, line 165):

“The main analyses involved Cox proportional hazards ~~logistic~~ regression modeling.”

Abstract:

“The association with thrombotic events remained in adjusted models (HR~~OR~~ [95%CI] per unit increase, 1.24 [1.04-1.47]~~1.33 [1.05-1.67]~~ for sTM; 1.07 [1.03-1.10] ~~1.16 [1.06-1.32]~~ for D-dimer). The adjusted regression model that included both biomarkers, sTM and D-dimer, improved significatively (AUC ~~75~~73%, sensitivity ~~72~~77% and specificity ~~72~~65% for TE diagnosis; p = 0.~~011~~007) the predictive capacity of the same model without sTM.”

Results (line 263):

“The association of higher sTM and D-dimer levels with thrombotic events remained in adjusted models (HR [95%CI] per unit increase, 1.24 [1.04-1.47]~~1.33 [1.05-1.67]~~ for sTM; 1.07 [1.03-1.10] ~~1.16 [1.06-1.32]~~ for D-dimer) (Figure 2).”

Results (line 274-281):

“In univariate models, the discriminative ability of both parameters for predicting thrombotic events was significant: sTM had an ~~OR~~ HR [95% CI] of 1.31~~1.38~~ [1.~~14~~13-1.~~69~~53 (p<0.001); sensitivity (se) = ~~70~~64%, specificity (sp) = ~~63~~70%] and D-dimer had an ~~OR~~ HR of 1.~~75~~08 [1.~~08~~05-~~2.83~~1.11 (p<0.001); se = ~~83~~67%, sp = ~~58~~74%]. At a cut-off point of 0.2, the adjusted regression model including both variables (sTM and D-dimer) showed higher specificity and significantly improved predictive capacity (AUC = ~~75~~73%, p = 0.~~011~~007; se = ~~72~~77% and sp = ~~72~~65%) compared with the same model without sTM (AUC = ~~72~~66%; se = ~~87~~66% and sp = ~~54~~60%) (Figure 3).”

Figure 2:

**Figure 2. Multivariate ~~logistic~~ Cox proportional hazards regression analysis of baseline predictors for 28-day thrombotic events in the propensity score cohort.**

**Figure 2 legend:** ~~HROR~~, Hazard~~Odds~~ Ratio; CI, confidence interval; RT-PCR, reverse transcriptase polymerase chain reaction. The figure presents adjusted ~~O~~HRs with 95% CIs for baseline predictors included in the multivariate model: soluble thrombomodulin (sTM), D-dimer, interleukin-6, and RT-PCR cycle threshold. The cohort was also matched by propensity score for age, sex, Charlson comorbidity index, and WHO COVID-19 severity score.

Figure 3:

Figure S5:

**Supplementary Figure S5. Multivariate Cox proportional hazards ~~logistic~~ regression analysis of baseline predictors for 28-Day venous thrombotic events in the propensity score cohort.**

~~OR~~HR, ~~odds~~ Hazard ratio; CI, confidence interval; RT-PCR, reverse transcriptase polymerase chain reaction for SARS-CoV-2. ~~P-value for s-Thrombomodulin = 0.051~~.

**6.- Figure 3: sTM mono is lacking – any difference to sTM+D-Dimer?**

Response: We appreciate this suggestion, as it has allowed us to further refine our presentation of the results. We have updated Figure 3 to include the ROC curve for sTM alone. As shown, the area under the curve (AUC) for sTM alone is 64.9 (95% CI: 55–75), which is lower than the AUC for D-dimer alone (66.4, 95% CI: 56–77) and significantly improved in the combined model (sTM + D-dimer), with an AUC of 72.5 (95% CI: 63–82). This analysis highlights the additive value of sTM when combined with D-dimer, reinforcing its utility in enhancing thrombotic risk prediction

Changes made in the manuscript: The revised version of Figure 3, and its title and legend is presented here.

**Figure 3.** Comparison of ROC curves for the ~~D-dimer~~ prediction of thrombotic events using D-dimer, soluble trombomodulin (sTM), and their combination~~with or without soluble thrombomodulin~~. Evaluation of the predictive performance of D-dimer alone, sTM alone, and the combination of D-dimer with sTM ~~in conjunction with soluble thrombomodulin versus D-dimer alone for the prediction of thrombotic events~~ in hospitalized COVID-19 patients.

**Figure 3 legend:** ROC, receiver operating characteristic; AUC, area under the ROC curve; s-TM, soluble thrombomodulin. In the legend, the first number represents sensitivity (%) and the second specificity (%). The cutoff value for the regression models in ~~both~~ all three cases was 0.2. The p-value for the comparisons between the combined model (D-dimer + sTM) and the models with D-dimer alone or sTM alone are 0.007 and 0.001, respectively. ~~Difference between the regression models was 0.011.~~

**7.- Figure legends are too short and do not explain what was done and what is seen. Worst example is suppl. Figure 3, I do not understand it.**

Response: We thank the reviewer for this valuable feedback regarding the figure legends. We have revised and expanded the legends to provide more detail on what was done and what is shown in each figure, including Supplementary Figure 3. We hope these changes improve clarity and address the reviewer’s concerns.

Changes made in the manuscript:

“**Figure 1. Boxplots illustrating soluble thrombomodulin (Panel a) and D-dimer (Panel b) plasma concentration in relation to mortality and thromboembolic events within 28 days.** Panels (a) and (b) show boxplots of soluble thrombomodulin (sTM) levels (ng/mL) in relation to thromboembolic events (a) and overall mortality (b). Panels (c) and (d) display D-dimer concentrations (µg/mL) in relation to thromboembolic events (c) and overall mortality (d). Statistical comparisons were performed using the Wilcoxon rank-sum test.”

**Figure 2 legend:** OR, odds Ratio; CI, confidence interval; RT-PCR, reverse transcriptase polymerase chain reaction. The figure presents adjusted HRs with 95% CIs for baseline predictors included in the multivariate model: soluble thrombomodulin (sTM), D-dimer, interleukin-6, and RT-PCR cycle threshold. The cohort was also matched by propensity score for age, sex, Charlson comorbidity index, and WHO COVID-19 severity score.

“**Supplementary Figure S3 legend**. SMD, standardized mean difference; Charlson, Charlson Comorbidity Index; WHO COVID-19, World Health Organization COVID-19 Ordinal Scale. This figure illustrates SMD for the variables used in the propensity score matching process: sex, age, Charlson comorbidity index, and WHO COVID-19 severity score. Open circles represent the SMD before matching ("All"), while filled circles represent the SMD after matching ("Matched"). The dashed line at 0.1 indicates the commonly accepted threshold for adequate balance. Values closer to 0 indicate better balance between cases and controls. The results demonstrate that the matching process significantly reduced imbalances in all variables, achieving acceptable balance across the matched cohort.”

**8. P8 line 197: no mortality risk of PAT is shown in suppl. Table 3.**

Response: Thank you for pointing this out. We understand the confusion regarding the mention of the Pulmonary Embolism Severity Index (PESI) in the manuscript and its relationship to Supplementary Table S3. The table includes the PESI categories for patients with pulmonary arterial thrombosis (PAT), but it does not explicitly show the corresponding mortality risk percentages for each category.

Changes made in the manuscript:

Results (“Characteristics and frequency of thrombotic events” section, line 202):

“The Pulmonary Embolism Severity Index (PESI) [26] indicated high risk of 30-day mortality (PESI category IV) in 28% of patients with PAT, and very high risk (PESI category V) in 17% (Supplementary Table S3).

Supplementary material document:

**Supplementary Table S3. Characteristics of thrombotic events.**

|  | **Value** | |
| --- | --- | --- |
| **Number of events** | 73 |  |
| **Days of symptoms**, median (Q1, Q3) | 5 | (3-8) |
| **TE type** | - |  |
| 1) ***Venous thrombosis*** | 56 | (77%) |
| 1.1) PAT | 46 |  |
| PESI category (Risk 30-day mortality^#^) | | |
| I (0-1.6%) | 3 |  |
| II (1.7-3.5%) | 10 |  |
| III (3.2-7.1%) | 12 |  |
| IV (4.0-11.4%) | 13 |  |
| V (10-24.5%) | 8 |  |
| CTPA features |  |  |
| Central | 11 |  |
| Peripheral | 35 |  |
| 1.2) DVT without PAT | 10 |  |
| 2) ***Arterial thrombosis**** | 17 | (23%) |

TE, thrombotic events; PAT, pulmonary arterial thrombosis; PESI, Pulmonary Embolism Severity Index (^#^Jacques Donzé, Grégoire Le Gal, Michael J Fine et al. Prospective validation of the Pulmonary Embolism Severity Index. A clinical prognostic model for pulmonary embolism. Thromb Haemost. 2008 Nov;100(5):943-8); Q1, first Quartile; Q3, third Quartile; CTPA, computed tomographic pulmonary angiography; DVT, deep venous thrombosis; *, Nine ischaemic strokes, six acute myocardial infarctions and two acute peripheral ischaemia cases.

**9. P10 line 223: D-Dimers did not correlate with Angoipoietin-2.**

Response: Thank you for pointing this out. You are correct that D-dimer does not correlate with angiopoietin-2, as shown in Supplementary Figure S4 (Pearson’s R = 0.007, p = non-significant). We apologize for the oversight in the text. To address this, we have revised the sentence to accurately reflect the data.

Changes made in the manuscript:

Results (“Analysis of coagulation plasma biomarkers” section, now in line 233):

The three coagulation parameters studied (sTM, Ang2 and D-dimer), showed a weak ~~but significant~~ positive correlation, with significant associations observed between sTM and both D-dimer and Ang2, while no correlation was observed between D-dimer and Ang2 each other (Supplementary Figure S4)~~, although we observed variable associations between the parameters and the study outcomes~~.

**10. Figure S4 – please find an easier way of labeling the plots with rho and p-values. Always give the values for p, no stars.**

Response: Thank you for your suggestion regarding the labeling in Supplementary Figure S4. We have revised the figure to replace the stars indicating significance levels with the exact p-values for clarity. Additionally, we have included both the rho and p-values directly within each plot for easier interpretation. We hope this updated version improves the readability of the figure.

Changes made in the manuscript:

Supplemental Figure S4:

**11. P10 line 234: give p-value for statement.**

Response: Thank you for your observation. The p-value for the comparison of Ang2 levels between omicron and non-omicron variants is p < 0.001. We have added this information to the revised manuscript for clarity.

Changes made in the manuscript.

Results (“Analysis of coagulation plasma biomarkers” section, line 246):

“… Ang2 levels were significantly higher in patients infected with the omicron variant (median [Q1, Q3] pg/mL, 734 [530, 1056] *vs.*360 [117, 837]; p<0.001).”

**12. P10 line 235 – mortality is a time-dependent event, what exactly is reported?**

Response: Thank you for your observation. The data presented in Figure 1 represent a univariate comparison of plasma concentrations (soluble thrombomodulin and D-dimer) between patients who survived and those who died within 28 days of hospital admission, without accounting for the timing of death. This analysis is intended to highlight crude differences in biomarker levels between the two groups.

Changes made in the manuscript:

Results (“Analysis of coagulation plasma biomarkers” section, now in line 247):

“Univariate analyses showed that ~~E~~elevated plasma concentration of sTM was significantly associated with mortality (median [Q1, Q3], 3.32 [2.16, 4.65] vs. 1.58 [1.11, 2.73] ng/…”

**13. Discussion: all arguments relate to a predictive value of the biomarkers, but this requires measurement at hospital admission.**

Response: We thank the reviewer for this insightful comment. We have addressed this point in our responses to comments 1 and 2, where we have unified the explanations and changes regarding the predictive or diagnostic nature of the study in the relevant sections of the manuscript to ensure consistency and clarity.
