# Supplementary material for: Enhanced prediction of thrombotic events in hospitalized COVID-19 patients with soluble thrombomodulin
Source: PLoS One. 2025 Mar 19;20(3):e0319666. doi: 10.1371/journal.pone.0319666 (PMC11922281; doi:10.1371/journal.pone.0319666)
Supplement: Attachment — Submitted filename: Reviewers response-2nd.docx [file pone.0319666.s004.docx]

Reviewers' comments: 
Reviewer's Responses to Questions

**REVIEWER #1: No Response**

**REVIEWER #2: Thank you for revising the manuscript.**

**I still have a problem with Figure 1, see my former comment 12.**

**Mortality is a time dependent event. For a KW-test looking at mortality in a binary way, the time frame for mortality has to be reported (e.g. 1-year mortality, then only include patients who died before or reached the landmark, take out patients with LFU before the landmark).**

Response: Thank you for this insightful comment. We appreciate the reviewer’s attention to this important aspect. Upon re-evaluating the data, we confirm that no patients were lost to follow-up before the 28-day study censoring date. Therefore, the figure and the analyses remain unchanged. However, to address this point and ensure clarity, we have added a note below the figure stating that all patients were accounted for within the 28-day follow-up period. We trust this addition provides the necessary clarification.

Changes made in the manuscript:

**Figure 1 legend:** All patients were accounted for within the 28-day follow-up period, with no losses to follow-up before the censoring date.

**Figure legends are better now, but Figure 1 shows a and b, the legend reports a-d.**

Response: Thank you for your observation. We have revised the figure legend to clarify that each panel includes data for thromboembolic events (left) and overall mortality (right). We hope this updated legend resolves the issue.

Changes made in the manuscript (The changes are highlighted for clarity):

**Figure 1. Boxplots illustrating soluble thrombomodulin (Panel a) and D-dimer (Panel b) plasma concentration in relation to 28-day mortality (right) and thromboembolic events (left) ~~within 28 days~~.** ~~Panels (a) and (b) show boxplots of soluble thrombomodulin (sTM) levels (ng/mL) in relation to thromboembolic events (a) and overall mortality (b). Panels (c) and (d) display D-dimer concentrations (µg/mL) in relation to thromboembolic events (c) and overall mortality (d). Statistical comparisons were performed using the Wilcoxon rank-sum test.~~
